# Asphyxia and Its Treatment

**Published:** 1856-05

**Authors:** 


*Asphyxia and its Treatment.*—Dr. Marshall Hall read a paper on this subject before the Harveian Society, December 6th, 1856.

He began by stating that, as the details of his investigation were before the Royal Humane Society, he could only place a brief abstract of them before the members of the Harveian Society.

His object was to show:

1st. That the blood during *circulation* becomes *self-poisoning*, chiefly by means of the carbonic acid formed.

2d. That the poison is, *pari passu,* eliminated by respiration.

3d. That, during suspended respiration, this carbonic acid poison accumulates in the blood.

4th. That *the special* means of obviating this effect, the *unicum remedium*, is to excite or imitate respiration.

5th. That every means of augmenting the circulation *without* simultaneous respiration, augments the formation of the carbonic acid poison, and consequently tends to destroy life.

6th. That the modes of inducing artificial respiration hitherto proposed, are nugatory and injurious, for the following reasons:

7th. 1. The posture in which this measure has been attempted being the supine, the tongue falls backward, carries with it the epiglottis, and closes the glottis against all *in*spiration.

8th. 2. That fluids accumulated in the fauces, either from external sources or by regurgitation from the stomach, operate in the same manner.

9th. 3. That the means of artificial respiration hitherto employed have been either of the nature of the *forcing*-pump or of the *suction*-pump.

10th. 4. That the former of these, besides having to overcome the impediment already described at the glottis, must necessarily be of force great enough to raise the ribs and carry down the diaphragm; and that such a force, as proved by Legallois and Leroy, may injure the delicate tissues of the lungs.

11th. 5. That the other mode of inducing respiration, by applying and removing pressure, is utterly inefficient, for the reason already mentioned, viz: the obstruction at the glottis.

12th. That there is ONE mode of inducing respiration which at once obviates all these difficulties, and proves all-efficient.

13th. That this consists: 1. In exchanging the *supine* for the PRONE position. 2. In inducing the movements of respiration by alternately allowing the weight of the subject to press on the thorax and abdomen by laying it on its face, and removing that pressure by raising it; this last effect being accomplished by raising the shoulders on the ilia as a centre, or by raising both shoulders and hips together by lifting; or, lastly, by turning the subject on the side.

14th. That these measures may be designated *prone*-respiration and *postural*-respiration respectively.

15th. That to these measures may be added gentle pressure, made on the posterior mark of the thorax and ribs, and its removal alternately.

16th. That whilst these measures are being adopted, others subsidiary, and injurious without them, may be superadded. Such are

17th. Rubbing the limbs upward with firm pressure, to promote warmth and the circulation; removing the wet clothes and replacing them with dry ones which the bystanders may supply, etc., etc.

18th. *Before* these measures are begun, every means of *exciting* respiration physiologically—such as irritating the nostrils and the fauces, dashing cold and hot water *alternately* on the face, etc., are to be fairly tried, the patient being first placed in the prone position for a moment, in order that any fluid present in the fauces may flow away.

19th. The face and chest may also, when the season is not inclement, be exposed to the breeze.

20th. But no time must be lost; the patient must be treated instantly, on the spot; messengers being despatched for medical aid, and every means of relief.

21st. It will be observed that the measures proposed are limited to such as may be *always* available; other questions, such as those that relate to the use of the warm bath, of galvanism, of the inhalation of oxygen, or of pure but dilute ammonia (to neutralize the carbonic acid blood-poison,) the transfusion of arterial blood, etc., etc., being left for another occasion.

Dr. Marshall Hall then observed that it would be obvious that the RULES for rescuing the patient affected by suspended respiration must undergo considerable modification. He added, that the investigation was also in the state of *progress;* and that he had especially to ascertain the precise *value* of each of our modes of treatment by careful *comparative* experiment, not again trusting to mere experience, as it is called, which, as it does not consist in *comparative* experiments, can lead to no accurate and definite results. For instance, the opinions in regard to the value of the warm-bath are not only different, but actually opposite, here and in France, judging from experience; whilst experiment and theory alike *prove* that it is and must be injurious, unless it be preceded and attended by respiration.—*Lancet, Dec.* 22, 1855, from *Amer. Journal Med. Sciences.*

